# Barriers to and Recommendations for Research Recruitment of Individuals Affected by Serious Illness

**DOI:** 10.1093/geroni/igab046.637

**Published:** 2021-12-17

**Authors:** Valeria Cardenas, Anna Rahman, Jenna Giulioni, Alexis Coulourides Kogan, Susan Enguidanos

**Affiliations:** 1 University of Southern California, University of Southern California, California, United States; 2 University of Southern California, Los Angeles, California, United States; 3 USC Division of Biokinesiology and Physical Therapy, Los Angeles, California, United States; 4 USC Keck School Of Medicine, Alhambra, California, United States

## Abstract

Researchers are encountering increasing challenges in recruiting participants for healthcare research. We conducted semi-structured individual interviews to identify participant barriers to research and recommendations for overcoming these challenges. We recruited 17 patients and eight caregivers who were approached to participate in a randomized control trial. We also recruited 31 primary care physicians. Using grounded theory, three researchers independently coded the transcripts and then met to compare codes and reconcile discrepancies. Themes from patient and caregiver interviews included time constraints, privacy concerns, lack of research familiarity, disconnect with research institution, self-perceived health status, and concerns with study randomization and repetitive questions. Physician-identified barriers focused on time constraints and study randomization. Patient and caregiver recommendations for study recruitment included various recruitment techniques. Physician recommendations were related to incentives. Although patients and caregivers prefer that their physicians recruit them for health-related research studies, physicians identified time constraints as a barrier to research involvement.

